# Comparative Analysis Between the EuroClonality-NGS Approach and the LymphoTrack^®^ Dx Assay for IG/TR Marker Screening in Lymphoid Leukemias: A Campus ALL Study

**DOI:** 10.3390/ijms27115115

**Published:** 2026-06-05

**Authors:** Irene Della Starza, Vittorio Bellomarino, Mariangela Di Trani, Luca Vincenzo Cappelli, Orietta Spinelli, Roberta Cavagna, Manuela Tosi, Carolina Terragna, Silvia Armuzzi, Valentina Robustelli, Barbara Taurisano, Alessandra Santoro, Domenico Salemi, Barbara Izzo, Santa Errichiello, Alessandra Galdiero, Roberta Visconti, Fabrizio Quarantelli, Alessandra Potenza, Matilde Francesca Caradonna, Matilde Marzorati, Grazia Fazio, Giovanni Cazzaniga, Deborah Cardinali, Francesca Kaiser, Ilaria D’Antuono, Sabina Chiaretti, Anna Guarini, Robin Foà

**Affiliations:** 1 Hematology, Department of Translational and Precision Medicine, Sapienza University, 00161 Rome, Italy; vittorio.bellomarino@uniroma1.it (V.B.); mariangeladitrani@gmail.com (M.D.T.); cappelli@bce.uniroma1.it (L.V.C.); deborah.cardinali@uniroma1.it (D.C.); francesca.kaiser@uniroma1.it (F.K.); ilaria.dantuonoo@gmail.com (I.D.); chiaretti@bce.uniroma1.it (S.C.); guarini@bce.uniroma1.it (A.G.); rfoa@bce.uniroma1.it (R.F.); 2 AIL Roma ODV, 00161 Rome, Italy; 3 Hematology and Bone Marrow Transplant Unit, Ospedale Papa Giovanni XXIII, 24127 Bergamo, Italy; ospinelli@asst-pg23.it (O.S.); rcavagna@fondazionefrom.it (R.C.); mtosi@asst-pg23.it (M.T.); 4 FROM Research Foundation, Ospedale Papa Giovanni XXIII, 24127 Bergamo, Italy; 5 IRCCS Azienda Ospedaliero-Universitaria di Bologna, Seràgnoli Institute of Hematology, 40138 Bologna, Italy; carolina.terragna@unibo.it (C.T.); silvia.armuzzi2@unibo.it (S.A.); valentina.robustell2@unibo.it (V.R.); barbara.taurisano@studio.unibo.it (B.T.); 6 Department of Medical and Surgical Sciences, University of Bologna, 40138 Bologna, Italy; 7 Division of Hematology and Bone Marrow Transplantation, Ospedali Riuniti Villa Sofia—Cervello, 90146 Palermo, Italy; a.santoro@villasofia.it (A.S.); d.salemi81@gmail.com (D.S.); 8 Department of Molecular Medicine and Medical Biotechnologies & CEINGE Advanced Biotechnologies, University of Naples Federico II, 80131 Naples, Italy; barbara.izzo@unina.it; 9 CEINGE Advanced Biotechnologies, University of Naples Federico II, 80145 Naples, Italy; errichiello@ceinge.unina.it (S.E.); galdiero@ceinge.unina.it (A.G.); viscontir@ceinge.unina.it (R.V.); quarantelli@ceinge.unina.it (F.Q.); potenza@ceinge.unina.it (A.P.); 10 Tettamanti Center, Fondazione IRCCS San Gerardo Dei Tintori, 20900 Monza, Italy; matildefrancesca.caradonna@irccs-sangerardo.it (M.F.C.); matilde.marzorati@irccs-sangerardo.it (M.M.); grazia.fazio@unimib.it (G.F.); giovanni.cazzaniga@unimib.it (G.C.); 11 School of Medicine and Surgery, University of Milano-Bicocca, 20900 Milan, Italy

**Keywords:** acute lymphoblastic leukemia (ALL), Next-generation sequencing (NGS) technology, immunoglobulin (IG) and T-cell receptor (TR)gene rearrangements

## Abstract

Next-generation sequencing (NGS) clonality assessment has the potential to overcome the conventional limitations of PCR-based methods. This study aimed at comparing the performance and concordance of the EuroClonality-NGS approach and the LymphoTrack^®^ Dx assay for immunoglobulin (IG)/T-cell receptor (TR)-marker identification at diagnosis. Across four quality-control rounds, six Italian laboratories analyzed 23 acute lymphoblastic leukemia (ALL) and five chronic lymphocytic leukemia (CLL) cases. Overall, 171 rearrangements were identified; 80.7% were detected by both methods. Among shared targets, the concordance was 89.1%, with a higher agreement for IG (IGH 100%–IGK 92.1%) than for TR loci (TRG 78.9%–TRB 81.8%). Bland–Altman analysis indicated no statistically significant systematic bias between methods [mean bias 1.99% (95% CI: −5.29 to +1.31%)]. The Spearman correlation was ρ = 0.680 (*p* < 0.001). Discordances (10.9%) rarely yielded suitable sensitive PCR assays for disease monitoring (20%) and were never the sole marker in individual patients. In larger independent cohorts, similar rates of no-marker cases were observed (~4–5% in adults), with higher frequencies in T-ALL and no association with blast percentage (Spearman’s ρ = 0.447, *p* = 0.450 in adults and ρ = 0.700, *p* = 0.188 in childhood). These findings support the reliability of both methods for diagnostic screening, while highlighting locus-specific variability and the importance of multi-target identification.

## 1. Introduction

Clonal rearrangements of immunoglobulin (IG) and T-cell receptor (TR) genes can serve as universal molecular targets in lymphoid leukemias and are widely used to assess disease traces. Their identification is therefore critical for both diagnosis and subsequent minimal residual disease (MRD) monitoring, with profound therapeutic implications [1]. This is true for both acute lymphoblastic leukemia (ALL) and chronic lymphocytic leukemia (CLL), where MRD has become a crucial decisional tool for treatment adaptation and transplant allocation.

Although the PCR-based method is the gold-standard for IG/TR clone detection in ALL, in about 5–10% of cases this technique fails to identify suitable markers for MRD evaluation [2]. This failure is likely due to intrinsic technical limitations such as assay design and the small number of primer combinations used, compared to all the possible V-D-J gene families.

In recent years, several next-generation sequencing (NGS)-based approaches [3,4,5,6] have been developed to overcome the limits of the conventional and standardized methods [7].

NGS technology can be used to detect clone-specific IG/TR index sequences allowing billions of DNA bases to be read at the same time [7]. By using universal primers, it allows the monitoring of the majority of IG/TR gene rearrangements (also when subclonal), providing a picture not only of the residual leukemia level but also of the normal immune repertoire. Moreover, this method can improve the sensitivity (ranging from 10^−4^ to 10^−6^) [8,9,10] and specificity of the primary diagnosis [11,12,13] and can also monitor the response to treatment [14,15,16].

The NGS platforms available—such as Illumina^®^ NextSeq/MiSeq (Illumina, San Diego, CA, USA), SOLID^®^ and Ion Torrent^®^ (Thermo Fisher Scientific, Waltham, CA, USA) —can provide high-quality and accurate large datasets [17]. This massive scale of data also generates challenges in processing and analyzing, prompting new developments in bioinformatic and data management technologies [18]. Indeed, the importance and advantages of NGS technology have been demonstrated in various hematologic disorders [10,14,19,20,21].

The EuroClonality-NGS working group has provided multiple reports on the development and standardization of IG/TR-NGS assays, focusing on marker identification protocols for subsequent MRD analysis [15,22]. Additionally, the NGS LymphoTrack^®^ assay (Invivoscribe, San Diego, CA, USA) [23,24] has being increasingly used to study IG/TR gene rearrangements in the last few years.

The aim of this study was to perform a comparative analysis between the EuroClonality and LymphoTrack^®^ NGS approaches, focused on the identification of the IG/TR marker in lymphoid leukemias at the onset of the disease with the idea of evaluating the potential and limits of the two methods and their reproducibility.

## 2. Results

### 2.1. Identification of IG/TR Markers

The first part of the study was a direct comparison of the two NGS methods and was conducted on 28 diagnostic samples from 23 ALL and 5 CLL cases with an available IG/TR marker by PCR screening.

A total of 171 IG/TR rearrangements were identified across the 28 analyzed samples. Of these, 138/171 (80.7%) were detected by both NGS approaches (shared markers: IGH-VJ, IGK, TRG, TRB), whereas 33/171 (19.3%) were identified exclusively by the EuroClonality-NGS assay (IGH-DJ and TRD targets not covered by LymphoTrack^®^). In detail, 23.5% (40/171) were IGH-VJ, 22.2% (38/171) IGK, 22.2% (38/171) TRG, 12.9% (22/171) TRB, 2.3% (4/171) IGH-DJ, and 16.9% (29/171) TRD. The mean number of rearrangements per sample was 4.8. The IGH-DJ and TRD rearrangements detected by EuroClonality-NGS never represented unique rearrangements and were always associated with other targets within the same patient.

**Figure 1:**
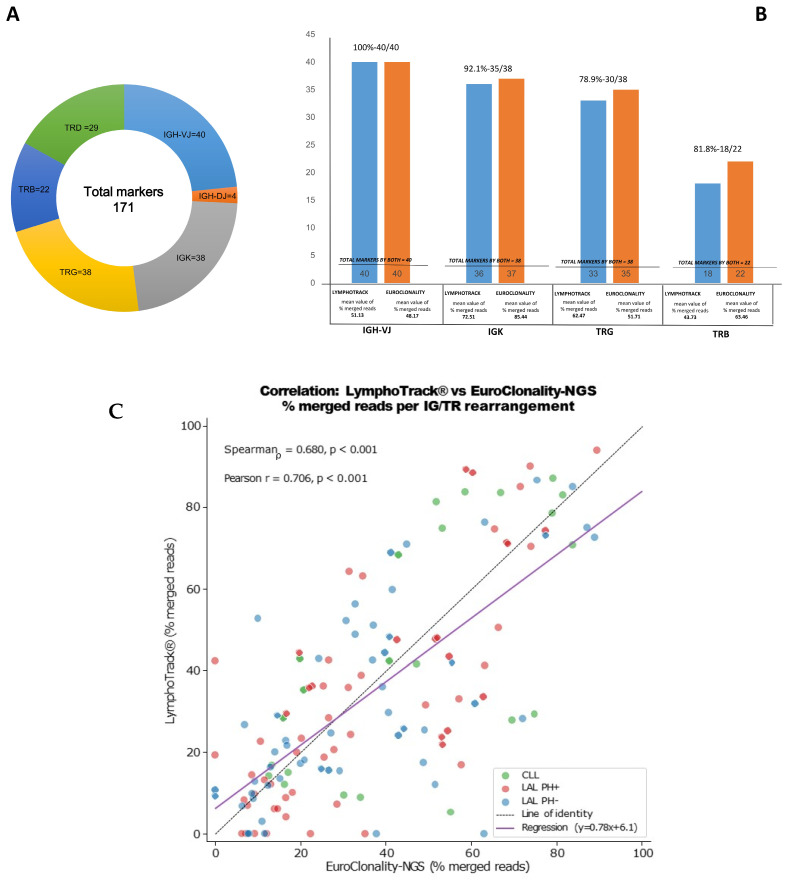
A diagram of IG/TR markers identified by the EuroClonality and LymphoTrack^®^ NGS approaches. (**A**) The number of IG/TR markers identified and the related distribution for each locus. (**B**) Concordance rate for the main markers identified by both methods: below each rectangle the number of targets and the mean value of % merged reads are reported proved by each NGS approach. (**C**) The correlation between LymphoTrack^®^ and EuroClonality-NGS for the quantification of IG/TR rearrangements. The dashed line represents the line of identity (y = x); the solid purple line represents the linear regression fit (y = 0.78x + 6.1). A significant positive correlation was observed between the two methods (Spearman ρ; Pearson r).

Concordance analysis was restricted to the 138 shared rearrangements. Stratified by locus, the concordance was 100% (40/40) for IGH-VJ (mean percentage of merged-reads: 51.13 by LymphoTrack^®^ and 48.17 by EuroClonality-NGS), 92.1% (35/38) for IGK (72.51 vs. 85.44), 78.9% (30/38) for TRG (62.47 vs. 51.71), and 81.8% (18/22) for TRB (43.73 vs. 63.46) (Figure 1). In the 5 CLL patients, the IGHV mutation rate (range 79–96.2%) did not affect the concordance.

Overall, 123/138 shared markers were consistently identified by both methods, corresponding to an overall concordance of 89.1%, whereas 15/138 (10.9%) were discordant and detected by only one approach. Discordances mainly involved TR loci (80.0%, 12/15; TRG/TRB), with the remaining cases affecting IGK (20.0%, 3/15); 11/15 were detected by EuroClonality-NGS and 4/15 by LymphoTrack^®^.

To better address this issue, we performed a Bland–Altman analysis on the 138 concordant IG/TR rearrangements identified by both methods across 28 patients, using the % merged reads as the quantitative variable (Figure 1C). The mean bias (LymphoTrack^®^−EuroClonality-NGS) was −1.99% (95% CI: −5.29 to +1.31%), indicating no statistically significant systematic bias between methods. The limits of agreement ranged from −40.4% to +36.4%, and 93.5% of data points fell within these limits. The Spearman correlation was ρ = 0.680 (*p* < 0.001). Locus-specific analysis showed the highest agreement for IGH (ρ = 0.802) and TRG (ρ = 0.779), while TRB showed the lowest correlation (ρ = −0.008, bias −14.65%), consistent with the greater technical complexity of this locus.

**Figure 2:**
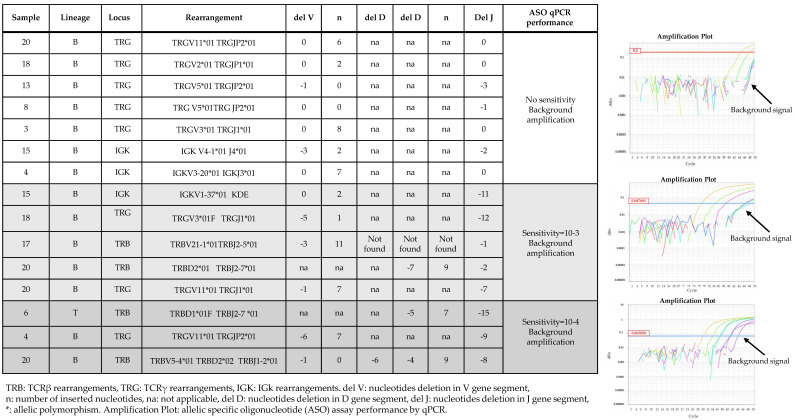
Discordant IG/TR rearrangements identified by EuroClonality and LymphoTrack^®^ NGS approaches in 9 adult ALL cases evaluated for clonality assessment.

To assess their suitability as MRD targets, allele-specific oligonucleotide (ASO)-qPCR assays were designed for all discordant rearrangements (n = 15). In 7/15 cases no suitable assay was obtained. Among the remaining eight, five showed non-patient-specific amplifications with a sensitivity of 10^−3^, whereas only 3/8 reached a sensitivity of 10^−4^, albeit with late background amplification (Figure 2). Notably, 6/15 (40.0%) discordant rearrangements lacked junctional deletions and exhibited short N-insertions.

Overall, only 3/15 (20%) discordant rearrangements (1 detected by LymphoTrack^®^ and 2 by EuroClonality-NGS) yielded a sufficiently sensitive assay for MRD monitoring.

Finally, to focus on the characteristics of the clono-sequences, V(D)J junctional features were analyzed across all identified targets. Median numbers of inserted and deleted nucleotides were 8.9 (range 0–23) and 5.9 (range 0–21) for IGH-VJ, 3.5 (0–20) and 4.6 (0–24) for IGK, 3.3 (0–17) and 4.2 (0–18) for TRG, and 4.8 (0–15) and 4.1 (0–13) for TRB rearrangements. For IGH-DJ and TRD rearrangements, median insertions and deletions were 8.7 (0–21) and 4.1 (0–7), and 6.3 (0–19) and 3.6 (0–27), respectively.

### 2.2. Analysis of No-Marker Cases: Correlation with Blast Percentage and Phenotype

Since the study cohort was based on marker-positive cases only, we performed internal analyses in a larger cohort of patients to evaluate the percentage of no-marker cases and its correlation with blast percentage (Table 1).

The Rome Lab used LymphoTrack^®^ to analyze 583 adult ALL cases (445 B, 130 T, and 8 Mixed-Phenotype Acute Leukemia-MPAL), and identified a total of 2436 rearrangements, with a mean value of rearrangements/sample of 4.2. The mean value of IG/TR marker cases was 4.8% (28/583), of which 14.3% (4/28) were B-ALL, 75% (21/28) T-ALL, and 10.7% (3/28) MPAL.

The Bergamo Lab used EuroClonality-NGS to analyze 514 adult ALL cases (339 B, 158 T and 17 MPAL) and identified a total of 2130 rearrangements, with a mean value of rearrangements/sample of 4.0. No IG/TR marker cases were 4.2% (22/514), of which 9.1% (2/22) were B-ALL, 77.3% (17/22) T-ALL, and 13.6% (3/22) MPAL.

The Monza Lab used EuroClonality-NGS to analyze 3480 pediatric ALL cases (3067 B, 402 T and 11 MPAL) and identified a total of 16133 rearrangements, with a mean value of rearrangements/sample of 4.7. No IG/TR marker cases were 1.7% (59/3480), of which 35.6% (21/59) were B-ALL, 61% (36/59) T-ALL, and 3.4% (2/59) MPAL (Table 1).

In the no-marker subgroup, no statistically significant association was observed between the blast percentage and no-marker frequency in both the pediatric and adult cohort (Spearman’s ρ = 0.447, *p* = 0.450 and ρ = 0.700, *p* = 0.188, respectively).

## 3. Discussion

The advancement of sequencing techniques has enabled the increasing use in lymphoid malignancies of NGS, which has shown to be a powerful tool for the identification of clonal rearrangements at disease onset [15]. NGS offers improved sensitivity, enabling the detection of minor rearrangements at diagnosis, helping to avoid missing clonal markers that could be responsible for relapse [6].

Here, we performed a comparative analysis between the EuroClonality and LymphoTrack^®^ NGS approaches for IG/TR marker identification in lymphoid leukemias to evaluate its potential, limitations, and interchangeability.

Firstly, we established a set of common analytical and interpretative parameters to ensure a methodologically correct comparison. In the study cohort, a total of 171 IG/TR markers from 28 patients (13 ALL Ph-, 10 ALL Ph+, 5 CLL) were identified: 80.7% were detected by both methods (IGH-VJ, IGK, TRG, TRB) and 19.3% by the EuroClonality-NGS approach (IGH-DJ and TRD) only. The overall concordance was 89.1%. Concordance was higher for IG rearrangements (92.5% and 92.1% for IGH and IGK, respectively), than for TR rearrangements (78.9% and 81.8% for TRG and TRB). The discrepancies may have technical and biologic reasons:

1. There are differences in the design of the two assays: different methods “see” the clonotype differently, with non-identical sensitivities and technical biases (bioinformatics interpretation, clustering algorithms, definition of “dominant clone” may differ between platforms).
2. Some TCR loci are notoriously more prone to generating analytical discordances than others: TRG (TCRγ) is easier to analyze but more prone to false positives/pseudoclonality; TRB (TCRβ) is biologically more complex, but often more specific and informative. The TRG generates more discordance because it has a limited repertoire. The number of V-J combinations is relatively small, and therefore reactive expansions may appear clonal, or poor samples may produce pseudoclonality. In NGS, the TRG often exhibits physiologic oligoclonality. Different pipelines may classify the same pattern differently. Therefore, the TRG is one of the loci most vulnerable to inter-analyte or inter-laboratory discordance.

TRB often shows better concordance with the actual clonal architecture and shows fewer false positives, but the locus is much more complex: it has a greater combinatorial diversity, a broader genomic structure, and can present multiple rearrangements. Technically, discrepancies may be due to an amplification bias with the risk of allelic dropout. Therefore, one method may miss a clone, another may not. TRB may present fewer false positives than TRG, but more technical false negatives.

The mean number of rearrangements per sample detected by both methods (4.8) was consistent with the 5.2 mean reported by Brüggemann et al. [15]. Supporting this observation, large-cohort analyses revealed mean rearrangements/sample of 4.2 (583 adult ALL, LymphoTrack^®^, Rome, Italy) and 4.0 (514 adult ALL, EuroClonality-NGS, Bergamo, Italy).

The IGH-DJ and TRD rearrangements detected only by EuroClonality-NGS were never detected as unique rearrangements in this study. Similarly, as reported on 211 clonal IG/TR gene rearrangements from 58 childhood B-ALL cases (detected by standard PCR), only 1 target belonging to incomplete TRD rearrangements (0.5%) resulted in unique rearrangement [25]. This finding suggests that the absence of these rearrangements in the LymphoTrack^®^ assay is unlikely to compromise the identification of suitable MRD markers in most clinical scenarios, particularly when multiple targets are available.

Since the characteristics of each IG/TR clono-sequence influence their suitability as MRD marker, we analyzed junctional V(D)J segmentation. Focusing on discordant markers (15/138, 10.9%), we attempted to design ASO-qPCR assays and test their performance: only 3/15 (20%) assays with a sensitivity of 10^−4^ were obtained; 1 marker was detected by LymphoTrack^®^ and 2 by EuroClonality-NGS. Of note, these rearrangements never resulted in a sole marker in any patient.

This suggests that these discordances do not represent major markers for the selection of patient-specific qPCR MRD testing, supporting the evidence of the comparability in the target detection between the two approaches. However, it is important to emphasize that discordant or missing markers have significant clinical relevance, especially for MRD assessment, as one method compared to another may not cover some rearrangements, may fail to detect subclones, or may miss evolutionary markers. Therefore, the same leukemic population may appear different in the two systems, and residual leukemic disease may be underestimated, with the risk of false-negative results and a consequent impact on prognostic stratification and therapeutic decisions. This underscores the importance of detecting more than one target, even when planning the use of NGS-based approaches for MRD analysis.

The failure to identify a MRD marker represents a significant issue: to address the limitation of a marker-positive study cohort, we performed additional analyses in larger independent datasets. Comparable rates of no-marker cases were observed between the two approaches in adult ALL, while lower rates were found in pediatric cohorts, likely reflecting the biologic differences between disease subtypes. The higher frequency of no-marker cases in T-ALL and the lack of association with the blast percentage suggest that intrinsic features of IG/TR rearrangements, rather than disease burden, may influence marker detectability.

Despite differences in the design of the two assays (EuroClonality-NGS is associated with a standardized workflow developed in an academic context, whereas LymphoTrack^®^ is a commercial kit-based assay), the two methods showed similar results in terms of their rate of target detection/sample, VDJ segmentations analysis, and percentage of no-marker cases. Moreover, in CLL patients the concordance between the two methods was not affected by IGHV status.

The superiority of the NGS approach in comparison to standard techniques in lymphoid malignancies has already been demonstrated [4,14,15,20,25,26,27,28,29]. Furthermore, despite its intrinsic complexity, NGS proved faster, more efficient and less laborious compared to standard workflow procedures [14,15,16]. However, it involves major costs, mainly determined by the number of genes investigated and the type of cartridge and consumables needed.

A cost comparison is difficult to carry out because it depends heavily on the number of loci, throughput, platform (MiSeq vs. Ion Torrent), automation, in-house bioinformatics, and diagnostic clonality tests vs. MRD analyses. However, realistic estimates can be made and for the same genes and the sample numbers per cartridge, LymphoTrack^®^ was generally more expensive than EuroClonality-NGS [(PCR reagents/Library prep per sample, sequencing = 350–650 €) vs. (PCR reagents/Library prep per sample, sequencing = 220–450 €), respectively]. This evaluation does not include technicians and instrument depreciation.

In an economic impact evaluation, for small- to medium-sized laboratories, LymphoTrack^®^ tends to be faster to implement and less dependent on bioinformatics expertise, while for large centers/reference labs EuroClonality-NGS can become cost-effective and more informative [e.g., EuroClonality-DNA capture approach]. Nevertheless, faced with a higher cost for the same time analysis (5–6 days), the EuroClonality-NGS workflow is reportedly more complex than the LymphoTrack^®^ approach [15,23].

These data are preliminary. The primary comparative analysis was performed on a relatively small cohort and restricted to cases with at least one detectable marker by conventional PCR screening, which may introduce selection bias, making differences emerge less clearly. Nevertheless, from the cohort of positive cases we did not exclude more informative borderline cases with low tumor burden (difference in blast percentage), incomplete rearrangements, or strong somatic hypermutation (CLL samples) that could have affected the agreement between the methods. In addition, the study focused on marker identification at diagnosis and further investigations are needed to increase the number of samples and to determine whether the observed concordance is maintained during longitudinal MRD monitoring.

## 4. Materials and Methods

### 4.1. NGS Marker Screening Analysis

Across four quality-control rounds from 2023 to 2025, six Italian laboratories (Rome, Bergamo, Bologna, Palermo, Naples and Monza) evaluated the comparability and reproducibility of the EuroClonality two-step approach and the Invivoscribe LymphoTrack^®^ Dx MiSeq assay for NGS-based IG/TR marker identification. The analyses were performed as previously reported [15,23,24].

All laboratories are members of the EuroMRD Consortium for Ph- and Ph+ ALL MRD analysis (http://www.euromrd.org, accessed on 9 April 2026).

At diagnosis, genomic DNA extracted from the mononuclear BM cells of 13 Ph-ALL patients (8 B-lineage and 5 T-lineage enrolled in the GIMEMA 1913 protocol), 10 Ph + ALL patients enrolled in the GIMEMA 2820 protocol (total ALL, n = 23), and 5 CLL cases referred to the Hematology Center of “Sapienza” University of Rome underwent screening for IGH-VJ, IGK-VJ-Kde, intron-Kde, TRG, TRB-VJ, and TRB-DJ gene rearrangements by both methods. IGH-DJ and TRD gene rearrangements were screened only by EuroClonality-NGS test, since the LymphoTrack^®^ approach lacks assays for these rearrangements.

The choice of sample was based on the availability of the material, its cellularity (range 3770–97,300 WBC), the blasts percentage (range 3–92%), and different percentages of IGHV mutations for CLL samples (range-79–96.2%).

CLL cases were included in the study with the aim of evaluating how much a mutated sequence affected the performance of the two NGS approaches in identifying the molecular marker.

The study was performed using the Illumina MiSeq (Illumina, San Diego, CA, USA) and Ion Torrent (Thermo Fisher Scientific, Waltham, CA, USA) platforms [30,31]. Data analysis was performed using the ARResT/Interrogate bioinformatics platform [22] for EuroClonality-NGS and LymphoTrack Dx Software [https://invivoscribe.com, accessed on 13 April 2026] for LymphoTrack^®^ respectively.

Additional analyses and validations were performed with the IMGT^®^ V-QUEST tool [International ImMunoGeneTics information system^®^—https://www.imgt.org, 13 April 2026].

A summary of the two NGS methods is reported in Table 2 and in Supplementary Table S1.

For discordant cases between the two methods, ASO-qPCR assays were designed according to EuroMRD guidelines [32,33] to evaluate the specificity of the rearrangements and determine whether they could represent true MRD markers.

### 4.2. Interpretative Criteria for Defining Clonality Status

To define the degree of clonality concordance, a cut off ≥5% and a number of reads of 10,000 per locus was established; both methods evaluated the clone percentage within the amplification pool.

Samples were considered oligoclonal if 3 or more clonotypes had a frequency of >5% for each rearrangement.

If no reads had a frequency >5%, the sample was considered polyclonal.

Concordance analysis was performed for each locus/target; the main markers (IGH-VJ, IGK, TRG, TRB) were considered for the comparison.

### 4.3. Statistical Analysis

Concordance between EuroClonality-NGS and LymphoTrack^®^ was evaluated as the proportion of shared rearrangements yielding consistent clonality calls based on pre-defined interpretative criteria, calculated separately for each IG/TR locus, and overall across the 138 targets amenable to direct comparison.

Agreement between the two methods for the quantitative variable of interest (percentage of merged reads per rearrangement) was assessed by Bland–Altman analysis. The mean bias and its 95% confidence interval (CI) were calculated as the mean of the differences (LymphoTrack^®^ − EuroClonality-NGS) ± 1.96 standard deviations (SD), defining the limits of agreement (LoA). Confidence intervals for the bias and LoA were derived using the standard error of the mean difference and the standard error of the limits, respectively, with a two-sided t-distribution critical value at α = 0.05. The proportion of data points falling within the LoA was reported. Bland–Altman analysis was performed both globally across all 138 shared rearrangements and stratified by locus (IGH, IGK, TRG, TRB).

The correlation between the two methods was assessed using Spearman’s rank correlation coefficient (ρ) and Pearson’s r, both reported with two-sided *p*-values.

For the analysis of no-marker cases in the independent validation cohorts, the association between blast percentage at diagnosis and no-marker frequency was evaluated by Spearman’s rank correlation between blast percentage bin midpoints and the number of no-marker cases per bin, performed separately in the pediatric (Monza) and adult (Roma/Bergamo) cohorts. Blast percentage distributions between the pediatric and adult no-marker subgroups were compared using the Mann–Whitney U test. A *p*-value < 0.05 was considered statistically significant for all analyses. All statistical analyses were performed using Python (version 3.12.3) with the SciPy and pandas libraries.

## 5. Conclusions

In summary, our results showed that the methodologic and technical differences between EuroClonality and LymphoTrack^®^ NGS approaches do not affect the reproducibility between the two methods, showing a good analytical comparability in IG/TR marker identification, supporting their reliability for diagnostic applications.

However, since analytical comparability is a statistical concept, while clinical interchangeability is a decision-making concept, patient-centered, and dependent on the clinical context and the cutoffs used, two methods may be very well correlated, but not clinically interchangeable if they generate different decisions.

So, to demonstrate their true interchangeability, it will be necessary to increase the number of samples analyzed and extend the analysis to longitudinal MRD follow-up samples as well.

Furthermore, the choice of one method over the other will depend on individual institutions’ factors, and some laboratory constraints and priorities such as automation, skilled personnel, turnaround times, and clinical and technical support could make one method more attractive than the other.

## Figures and Tables

**Table 1:** No-marker cases analysis by the EuroClonality and LymphoTrack^®^ NGS approaches in a large cohort of childhood and adult ALL.

Lab	Method	Patients	IG/TR rr * Identified	Marker Positive %	Mean rr/Sample *	No IG/TR Marker %	Immunophenotype Lineage of No-Marker Cases
Rome	LymphoTrack^®^	583 adult ALL (445 B, 130 T, 8 MPAL)	2436	95.2 (555/583)	4.2	4.3 (28/583)	14.3% (4/28) B-ALL 75% (21/28) T-ALL 10.7% (3/28) MPAL
Bergamo	EuroClonality-NGS	514 adult ALL (339 B, 158 T, 17 MPAL)	2130	95.7 (492/514)	4.0	4.2 (22/514)	9.1% (2/22) B-ALL 77.3% (17/22) T-ALL 13.6% (3/22) MPAL
Monza	EuroClonality-NGS	3480 pediatric ALL (3067 B, 402 T, 11 MPAL)	16,133	98.3 (3421/3480)	4.7	1.7 (59/3480)	35.6% (21/59) B-ALL 61% (36/59) T-ALL 3.4% (2/59) MPAL

* rr = rearrangements.

**Table 2:** Technical comparison between two methods.

Feature	EuroClonality-NGS	LymphoTrack^®^
Method	Amplicon-based NGS approach with a standardized workflow for IG/TR marker identification, validated across many experts European laboratories.	A commercial kit-based assay using amplicon-based NGS for the targeted sequencing of IG and TCR loci (IGH-VJ, IGK-VJ-Kde, intron-Kde, TRG, TRB-VJ, TRB-DJ).
	The method offers a broad, integrated analysis for detecting B- and T-cell clonality (IGH-VJ, IGH-DJ, IGK-VJ-Kde, intron-Kde, TRG, TRD, TRB-VJ, TRB-DJ).	The method requires complementary assays for full analysis, such as incomplete rearrangements (IGH-DJ and TRD).
Sensitivity	High sensitivity Ranged from 10^−4^ to 10^−6^ (depending on amounts of DNA analyzed)	High sensitivity Ranged from 10^−4^ to 10^−6^ (depending on amounts of DNA analyzed)
Turnaround Time	Reduced from traditional methods with a faster workflow.	Substantially reduced compared to traditional methods with kits allowing for a faster workflow.
Workflow	Wet lab: 3 days Sequencing: 36 h * Data analysis:1 day	Wet lab: 3 days Sequencing: 36 h * Data analysis:1 day
Sequencing	Illumina MiSeq platform Ion Torrent platform (ref. [30,31])	Illumina MiSeq platform Ion Torrent platform (ref. [30,31])
Bioinformatic tool	ARResT/Interrogate bioinformatics platform (ref. [22])	LymphoTrack Dx Software
Applicability	High applicability (>95%) Potential to identify clonal evolution Provides information on background repertoire of B and T cells	High applicability (>95%) Potential to identify clonal evolution Provides information on background repertoire of B and T cells

* Time is calculated as 2 × 250 paired-end sequencing on MiSeq Platform (Illumina).

## Data Availability

The original contributions presented in this study are included in the article/Supplementary Material. Further inquiries can be directed to the corresponding author.

## Supplementary Materials

The following supporting information can be downloaded at: https://www.mdpi.com/article/10.3390/ijms27115115/s1.

## Abbreviations

The following abbreviations are used in this manuscript:

NGS	Next-generation sequencing
IG	Immunoglobulin
TR	T-cell receptor
MRD	Minimal Residual Disease
ALL	Acute lymphoblastic leukemia
CLL	Chronic lymphocytic leukemia
MPAL	Mixed-Phenotype Acute Leukemia
ASO	Allele-Specific Oligonucleotide
qPCR	Quantitative Polymerase Chain Reaction
CI	Confidence Interval
SD	Standard Deviation
LoA	Limit of Agreement
