# Supplementary material for: Comparative Analysis Between the EuroClonality-NGS Approach and the LymphoTrack® Dx Assay for IG/TR Marker Screening in Lymphoid Leukemias: A Campus ALL Study
Source: Int J Mol Sci. 2026 Jun 5;27(11):5115. doi: 10.3390/ijms27115115 (PMC13256919; doi:10.3390/ijms27115115)
Supplement: Supplementary file 1 [file ijms-27-05115-s001.zip › Supplementary Table S1.pptx]

## Slide 1
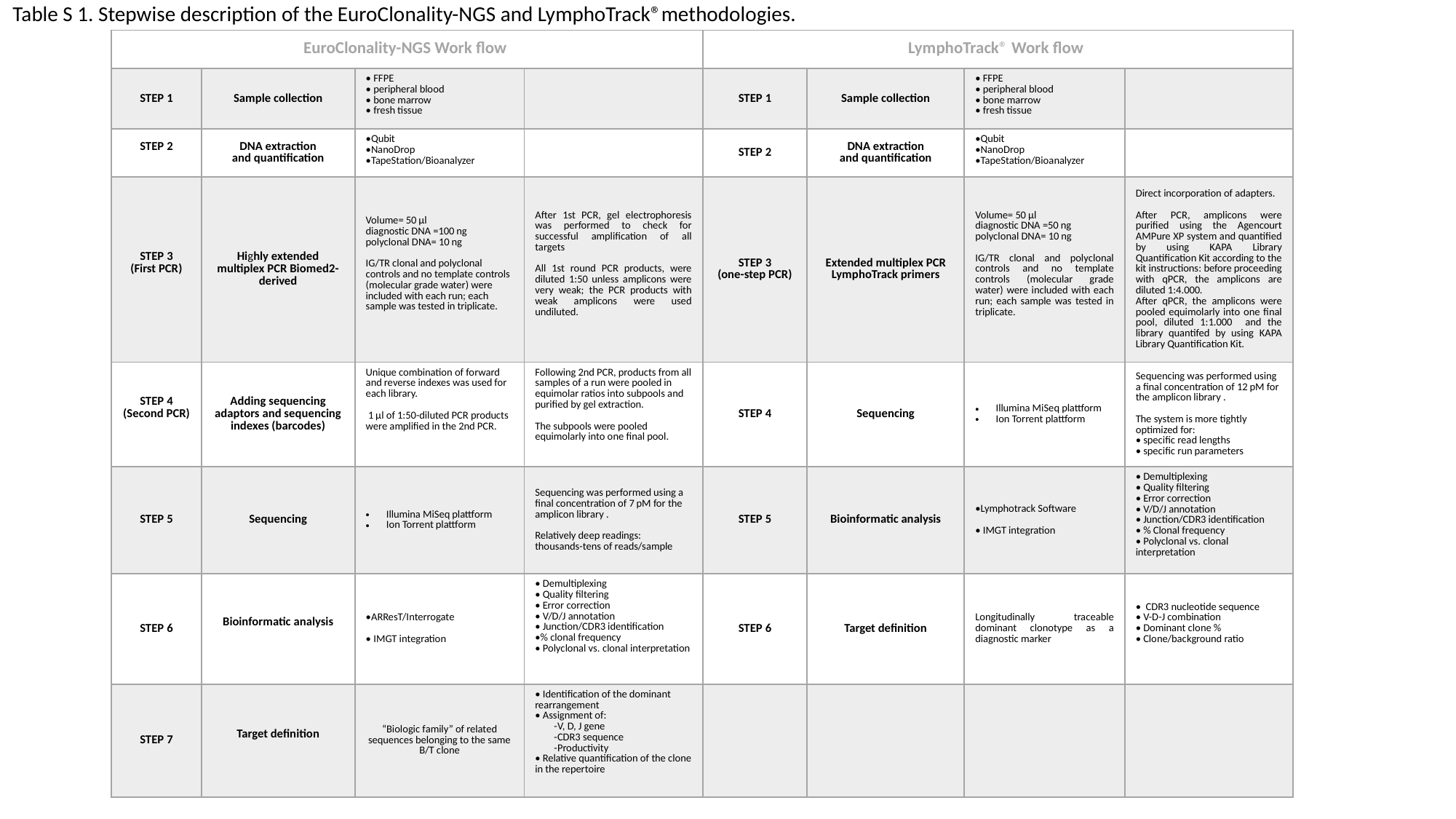

Table S 1. Stepwise description of the EuroClonality-NGS and LymphoTrack®methodologies.
| EuroClonality-NGS Work flow | | | | LymphoTrack® Work flow | | | |
| --- | --- | --- | --- | --- | --- | --- | --- |
| STEP 1 | Sample collection | • FFPE • peripheral blood • bone marrow • fresh tissue | | STEP 1 | Sample collection | • FFPE • peripheral blood • bone marrow • fresh tissue | |
| STEP 2 | DNA extraction and quantification | •Qubit •NanoDrop •TapeStation/Bioanalyzer | | STEP 2 | DNA extraction and quantification | •Qubit •NanoDrop •TapeStation/Bioanalyzer | |
| STEP 3 (First PCR) | Highly extended multiplex PCR Biomed2-derived | Volume= 50 µl diagnostic DNA =100 ng polyclonal DNA= 10 ng IG/TR clonal and polyclonal controls and no template controls (molecular grade water) were included with each run; each sample was tested in triplicate. | After 1st PCR, gel electrophoresis was performed to check for successful amplification of all targets All 1st round PCR products, were diluted 1:50 unless amplicons were very weak; the PCR products with weak amplicons were used undiluted. | STEP 3 (one-step PCR) | Extended multiplex PCR LymphoTrack primers | Volume= 50 µl diagnostic DNA =50 ng polyclonal DNA= 10 ng IG/TR clonal and polyclonal controls and no template controls (molecular grade water) were included with each run; each sample was tested in triplicate. | Direct incorporation of adapters. After PCR, amplicons were purified using the Agencourt AMPure XP system and quantified by using KAPA Library Quantification Kit according to the kit instructions: before proceeding with qPCR, the amplicons are diluted 1:4.000. After qPCR, the amplicons were pooled equimolarly into one final pool, diluted 1:1.000 and the library quantifed by using KAPA Library Quantification Kit. |
| STEP 4 (Second PCR) | Adding sequencing adaptors and sequencing indexes (barcodes) | Unique combination of forward and reverse indexes was used for each library. 1 µl of 1:50-diluted PCR products were amplified in the 2nd PCR. | Following 2nd PCR, products from all samples of a run were pooled in equimolar ratios into subpools and purified by gel extraction. The subpools were pooled equimolarly into one final pool. | STEP 4 | Sequencing | Illumina MiSeq plattform Ion Torrent plattform | Sequencing was performed using a final concentration of 12 pM for the amplicon library . The system is more tightly optimized for: • specific read lengths • specific run parameters |
| STEP 5 | Sequencing | Illumina MiSeq plattform Ion Torrent plattform | Sequencing was performed using a final concentration of 7 pM for the amplicon library . Relatively deep readings: thousands-tens of reads/sample | STEP 5 | Bioinformatic analysis | •Lymphotrack Software • IMGT integration | • Demultiplexing • Quality filtering • Error correction • V/D/J annotation • Junction/CDR3 identification • % Clonal frequency • Polyclonal vs. clonal interpretation |
| STEP 6 | Bioinformatic analysis | •ARResT/Interrogate • IMGT integration | • Demultiplexing • Quality filtering • Error correction • V/D/J annotation • Junction/CDR3 identification •% clonal frequency • Polyclonal vs. clonal interpretation | STEP 6 | Target definition | Longitudinally traceable dominant clonotype as a diagnostic marker | • CDR3 nucleotide sequence • V-D-J combination • Dominant clone % • Clone/background ratio |
| STEP 7 | Target definition | “Biologic family” of related sequences belonging to the same B/T clone | • Identification of the dominant rearrangement • Assignment of: -V, D, J gene -CDR3 sequence -Productivity • Relative quantification of the clone in the repertoire | | | | |
